# Effect of nanostructured carbon coatings on the electrochemical performance of Li_1.4_Ni_0.5_Mn_0.5_O_2+_*_x_*-based cathode materials

**DOI:** 10.3762/bjnano.7.187

**Published:** 2016-12-09

**Authors:** Konstantin A Kurilenko, Oleg A Shlyakhtin, Oleg A Brylev, Dmitry I Petukhov, Alexey V Garshev

**Affiliations:** 1 Department of Chemistry, M. V. Lomonosov Moscow State University, 119991 Moscow, Russia; 2 Department of Materials Sciences, M. V. Lomonosov Moscow State University, 119991 Moscow, Russia

**Keywords:** carbon coatings, electrode materials, Li-ion batteries, nanocomposites, nanostructures

## Abstract

Nanocomposites of Li_1.4_Ni_0.5_Mn_0.5_O_2+_*_x_* and amorphous carbon were obtained by the pyrolysis of linear and cross-linked poly(vinyl alcohol) (PVA) in presence of Li_1.4_Ni_0.5_Mn_0.5_O_2+_*_x_*. In the case of linear PVA, the formation of nanostructured carbon coatings on Li_1.4_Ni_0.5_Mn_0.5_O_2+_*_x_* particles is observed, while for cross-linked PVA islands of mesoporous carbon are located on the boundaries of Li_1.4_Ni_0.5_Mn_0.5_O_2+_*_x_* particles. The presence of the carbon framework leads to a decrease of the polarization upon cycling and of the charge transfer resistance and to an increase in the apparent Li^+^ diffusion coefficient from 10^−16^ cm^2^·s^−1^ (pure Li_1.4_Ni_0.5_Mn_0.5_O_2+_*_x_*) to 10^−13^ cm^2^·s^−1^. The nanosized carbon coatings also reduce the deep electrochemical degradation of Li_1.4_Ni_0.5_Mn_0.5_O_2+_*_x_* during electrochemical cycling. The nanocomposite obtained by the pyrolysis of linear PVA demonstrates higher values of the apparent lithium diffusion coefficient, a higher specific capacity and lower values of charge transfer resistance, which can be related to the more uniform carbon coatings and to the significant content of sp^2^-hybridized carbon detected by XPS and by Raman spectroscopy.

## Introduction

LiNi_0.5_Mn_0.5_O_2_-based electrode materials [1] were proposed as a less expensive alternative to LiCoO_2_ for high energy density Li-ion batteries containing less toxic elements than cobalt. The reasonable combination of their electrochemical properties (*C* = 160–180 mAh·g^−1^ at *C*/10; *U* = 2.5–4.6 V) remains attractive until now [2–4]. Most of the studies deal with Li(Ni,Mn)O_2_ with equimolar amounts of nickel and manganese. The influence of the Ni/Mn ratio on the properties of these materials is discussed in [5]. One of the obstacles to the practical application of LiNi_0.5_Mn_0.5_O_2_ is related to the internal ion exchange (ion mixing) of Li^+^ and Ni^2+^ due to the very similar ionic radii. This process could be partially suppressed by the introduction of extra lithium into LiNi_0.5_Mn_0.5_O_2_. Extra lithium ions are usually located in the (Ni,Mn) sublattice with the formation of Li[Li*_x_*Ni_0.5−(_*_x_*_/2)_Mn_0.5−(_*_x_*_/2)_]O_2−δ_ [6–7]. The practical application of these materials is limited by the insufficient electronic conductivity of Li_1+_*_x_*(Ni,Mn)O_2_ materials [8] and their ability to catalyze the organic electrolyte decomposition at high potentials and currents [9–10].

The most common way of overcoming this problem is the modification of cathode materials by introducing additives and by depositing coatings that would suppress the interaction of electrolyte and the surface of particles. Various kinds of materials have been tested for surface modification, namely other cathode materials (LiMnPO_4_ [11], LiMn_2_O_4_ [12], LiCoO_2_ [13], LiNiO_2_ [14]) or simple binary compounds such as CaF_2_ [15], TiO_2_ [16], ZnO [17] and Al_2_O_3_ [18].

During the assembly of the lithium-ion cells, the cathode materials are always mechanically mixed with carbon black in order to enhance the electronic conductivity. However, this method of carbon introduction provides the contact only between the external surfaces of cathode materials aggregates, but it does not improve the coherence and hence the electrical conductivity inside the aggregates.

Both kinds of problems can be solved by the fabrication of conducting carbon coatings on the surface of Li(Ni,Mn,Co)O_2+_*_x_* particles [19–22]. Such coatings can be obtained by the impregnation of oxide powders with the solution or the melt of organic compounds accompanied by subsequent heat treatment in the absence of oxygen or at limited oxygen access [23–25]. These carbonaceous coatings improve the electrokinetic properties of cathode materials, which results in enhanced capacities at high discharge rates [26]. The variation in the electrochemical properties is governed by the increase in the electronic conductivity of the obtained coatings, which depends on both the organic precursor composition and the preparation conditions. Thus, the investigation of the influence of physicochemical properties of carbon coatings on the electrochemical properties of Li(Ni,Mn,Co)O_2+_*_x_*/C composites proves to be an important task. The choice of an organic precursor is an important challenge. In the case of Li(Ni,Mn)O_2_, it is rather complicated due to the high oxidizing ability of this material, which leads to its intensive interaction with both the products of the pyrolysis of organic compounds [27] and carbon [28]. As it has been shown before, the rate of this interaction could be significantly reduced by using some polymer precursors [28]. They allow for a solid conducting film consisting of pyrolysis products to be obtained at moderate temperatures (300–350 °С). Poly(vinyl alcohol) (PVA) could be one of such precursors. It eliminates water at temperatures higher than 230 °С, forming carbon residues with a system of conjugated double bonds [29–30].

In the present paper, the electrochemical properties of Li_1.4_Ni_0.5_Mn_0.5_O_2+_*_x_*/C composites are discussed, the preparation and characterization of which have been described before in detail [28,31]. The melts of linear or cross-linked PVA served as carbon source. We investigated the influence of composition and micro/nanomorphology of the carbonaceous coatings obtained by the pyrolysis of various kinds of PVA on the kinetics of lithium intercalation–deintercalation and the electrochemical properties of Li_1.4_Ni_0.5_Mn_0.5_O_2+_*_x_*/C composites.

## Results and Discussion

**Figure 1:**
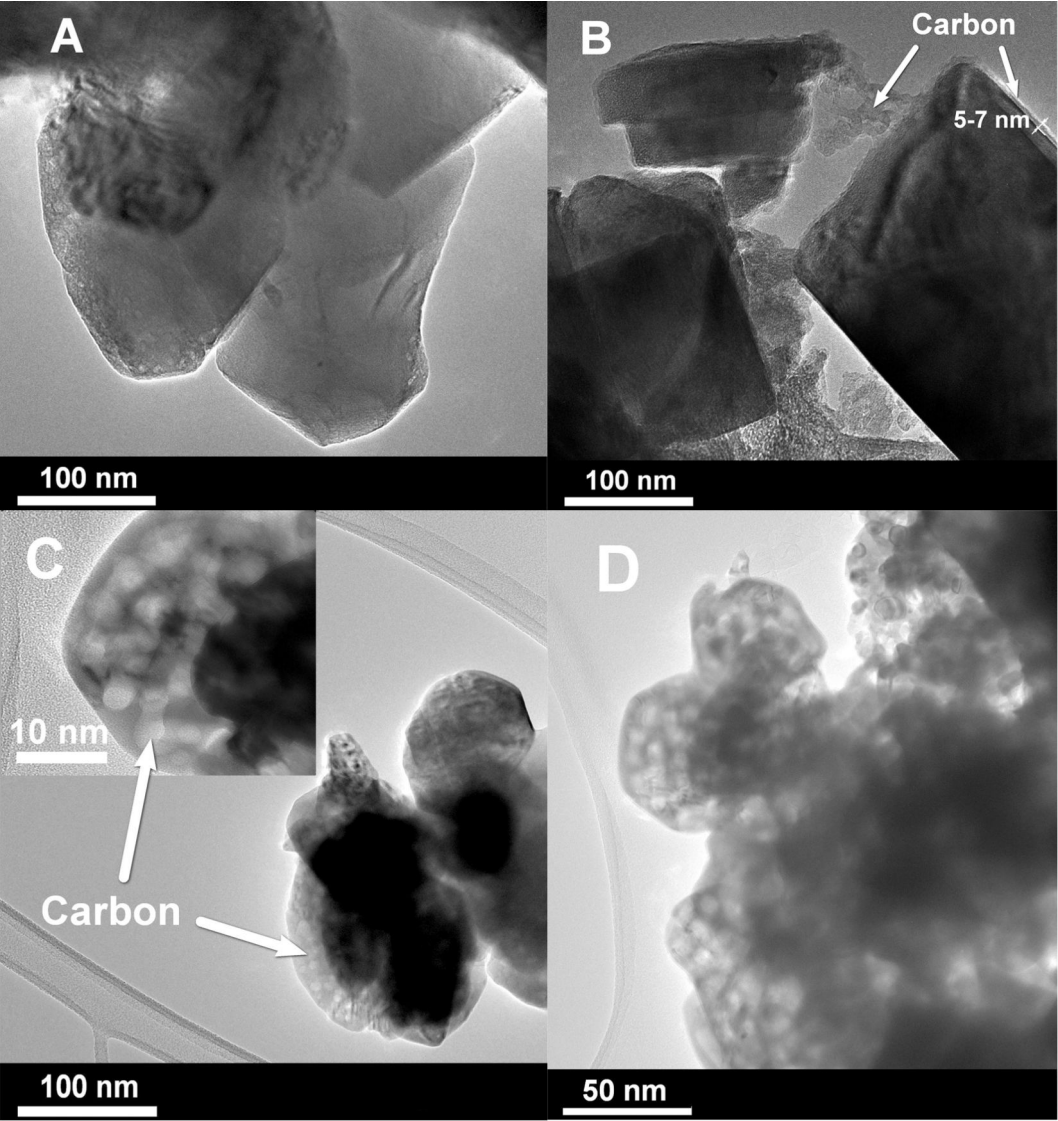
TEM micrographs: A) pure LNM; B) LNM + carbon (linear PVA); C) LNM + carbon (cross-linked PVA); D) carbon, obtained by the isothermal heat treatment of cross-linked PVA at 350 °С for 15 min in argon.

TEM micrographs of pure Li_1.4_Ni_0.5_Mn_0.5_O_2+_*_x_* (LNM) and LNM/C composites obtained by the pyrolysis of both linear and cross-linked PVA are shown in Figure 1. In the case of LNM/C composites obtained from linear PVA, 5–7 nm thick carbon coatings can be clearly observed on the particle surface as well as amorphous carbon bottlenecks between particles forming a consolidated 3D network (Figure 1B). Concerning the LNM/C composites obtained from cross-linked PVA, the continuous carbonaceous coatings on oxide particles are absent. The carbon is localized as separate islands on the boundaries of Li_1.4_Ni_0.5_Mn_0.5_O_2+_*_x_* particles (Figure 1C). The carbon obtained from cross-linked PVA has a sponge-like microstructure with nanometer-sized mesopores (Figure 1D).

**Figure 2:**
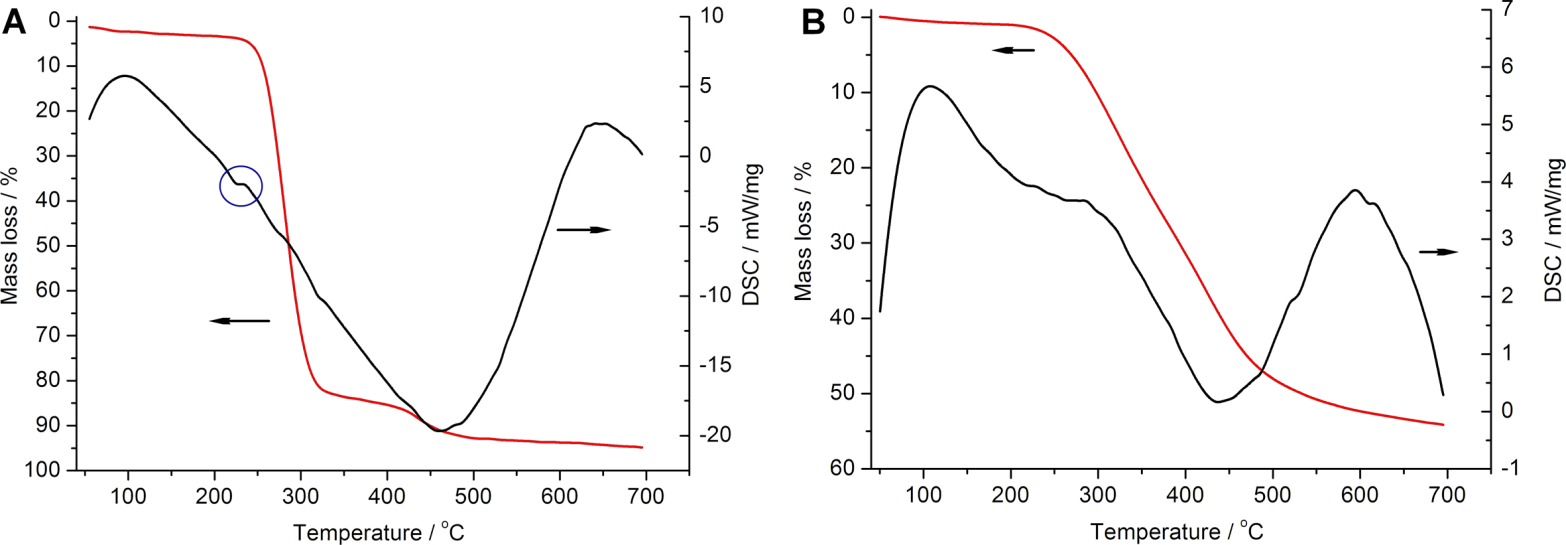
TG-DSC curves of the linear (A) and cross-linked (B) PVA in argon.

Thermogravimetry (TG) curves of the linear and cross-linked PVA demonstrate the intense pyrolysis of the linear PVA at 240–310 °C (Figure 2A) and the continuous decomposition process of the cross-linked PVA at 240–600 °C (Figure 2B). The latter shows a significantly higher thermal stability of the cross-linked polymer. The analysis of differential scanning calorimetry (DSC) curves allows one to identify a small “endo”-effect at 230 °C before the decomposition of the linear PVA (Figure 2A) that could be associated with its melting [32]. The corresponding effect is absent in the DSC curve of the cross-linked polymer (Figure 2B).

The reasons for the different localization of carbon in the samples can be associated with the observed features of the pyrolysis of precursors. Melting of linear PVA causes a wetting of LNM grains with the polymer melt followed by a relatively uniform pyrolysis of the thin polymer films on the surface of oxide crystallites. In the case of cross-linked PVA, the polymer pyrolysis tentatively occurs without preliminary melting. The pyrolysis of PVA particles allocated in the voids between LNM crystallites results in forming mesoporous particulates of amorphous carbon in the interparticular space.

**Figure 3:**
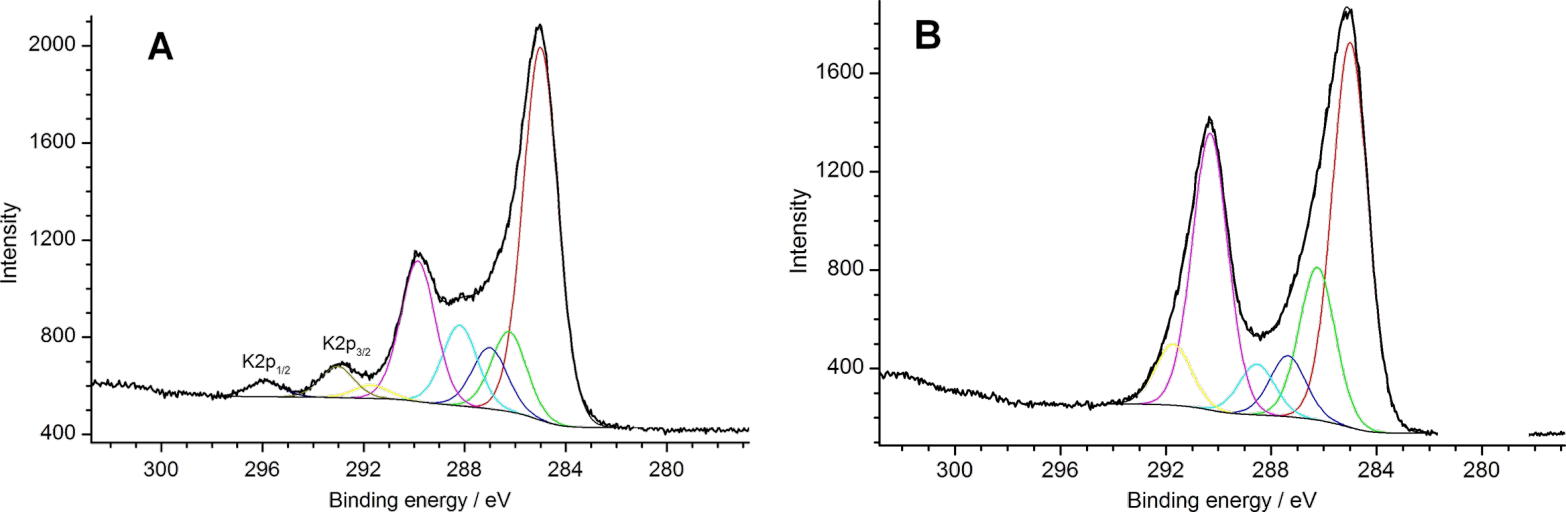
XPS spectra of C 1s of the LNM/C composite samples derived from the linear (A) and cross-linked (B) PVA.

Another difference between the pyrolysis products of linear and cross-linked PVA concerns their different chemical composition. According to the C 1s XPS spectra of carbon-coated LNM (Figure 3, Table 1), the pyrolysis products of linear PVA contain larger amounts of C–C and C=C bonds compared to the pyrolysis products of cross-linked PVA. This difference could be associated to the easier dehydration of linear PVA at 350 °C resulting in the formation of C=C bonds: –(CH_2_–CH(OH))*_n_*– → –(CH=CH)*_n_*– + *n*H_2_O compared to the complicated thermal decomposition of –(СН_2_–CH–O–CH–CH_2_)*_n_*– chains of cross-linked PVA.

**Table 1:** Fractions of C 1s XPS spectra components of the LNM/C composite samples derived from the linear and cross-linked PVA.

sample	fraction of C 1s components, %					

	285.0 eV	286.3 eV	287.2 eV	288.3 eV	290.1 eV	291.7 eV

	С=С (С–С)	C–O	C=O	O–C=O	–CO_3_^2−^	—

LNM + linear PVA	50.7	9.7	8.2	7.8	19.8	3.8
LNM + cross-linked PVA	35.9	15.5	9.2	5.1	28.1	6.2

**Figure 4:**
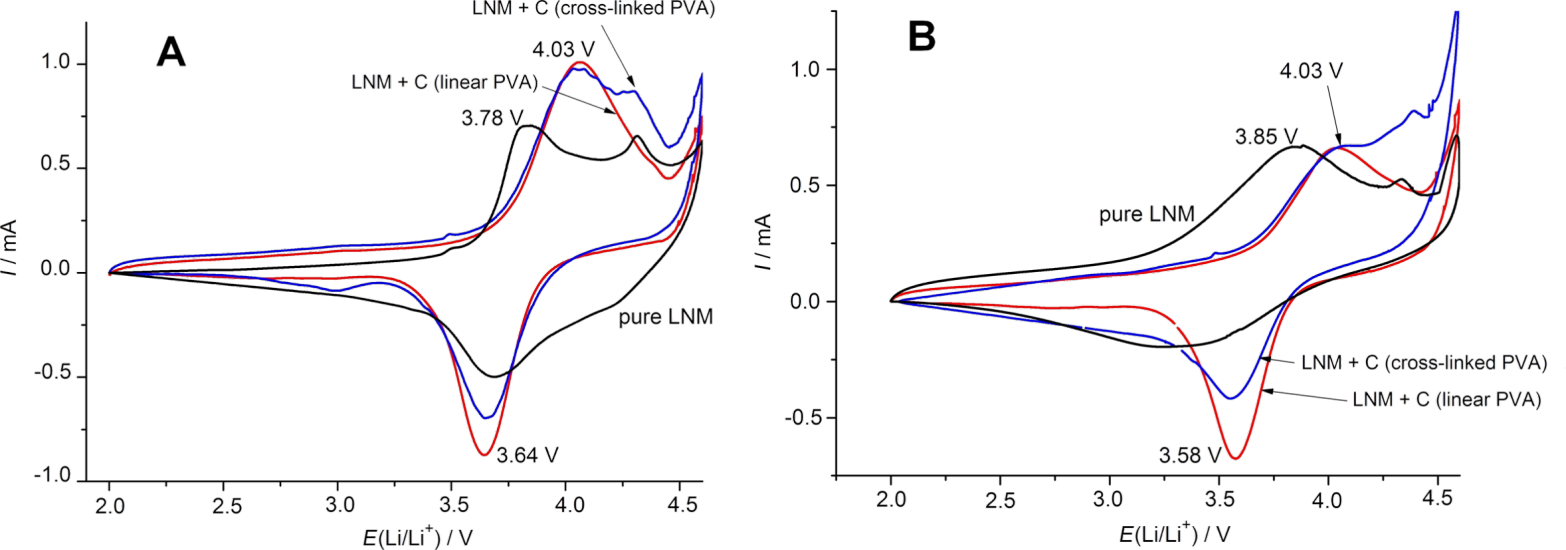
CVA curves of pure Li_1.4_Ni_0.5_Mn_0.5_O_2+_*_x_* and LNM/C nanocomposites at the 1st cycle (A) and after galvanostatic cycling (B) at discharge currents of 20–100 mA·g^−1^ (the 15th cycle). Potential scan rate is 50 µV·s^−1^.

The electrochemical properties of as-obtained nanocomposites were studied by cyclic voltammetry (CVA) to estimate the influence of carbon coatings on the lithium intercalation–deintercalation in Li_1.4_Ni_0.5_Mn_0.5_O_2+_*_x_* (Figure 4). Concerning the LNM/C composites, the cathodic peaks are substantially narrower than the ones for pure Li_1.4_Ni_0.5_Mn_0.5_O_2+_*_x_*. In the case of LNM/C composites, the lithium intercalation is almost completely finished at 3.64 V, but for pure Li_1.4_Ni_0.5_Mn_0.5_O_2+_*_x_* it takes place over a wider potential range. It means that the carbon nanocoatings enable the polarization decrease upon lithium intercalation [23]. After galvanostatic cycling at 20–100 mA·g^−1^, CVA curves were recorded at the 15th cycle (Figure 4В). In the case of pure Li_1.4_Ni_0.5_Mn_0.5_O_2+_*_x_*, the potential range of lithium intercalation expands and in the anodic region, the main lithium deintercalation peak shifts towards higher potentials (from 3.78 V at the 1st cycle to 3.85 V at the 15th cycle). This fact confirms a poorer reversibility of lithium insertion/extraction into the pure Li_1.4_Ni_0.5_Mn_0.5_O_2+_*_x_*.

The electrokinetic properties of LNM/C nanocomposite obtained by the pyrolysis of linear PVA are significantly better than the ones of the corresponding composite obtained by the pyrolysis of cross-linked PVA. In particular, after galvanostatic cycling at 20–100 mA·g^−1^, the cathodic peak at ca. 3.6 V is more pronounced and the range of lithium intercalation potentials is narrower (Figure 4). Apparently, this phenomenon could be ascribed to a more uniform carbon distribution and to the higher content of conductive sp^2^-hybridized carbon in the coatings obtained by pyrolysis of linear PVA compared to the pyrolysis of cross-linked PVA [28]. Another reason for the superior electrokinetic properties of the LNM/C nanocomposite obtained from linear PVA could be associated with the absence of the reversible structural transition in the partially delithiated Li_1.4_Ni_0.5_Mn_0.5_O_2+_*_x_* that could be indicated by the absence of anodic peak at 4.3 V [33] clearly visible for the other two samples. The appearance of this transition, which causes additional internal stress during cycling in the more polarized cathode materials, could be promoted by a less homogeneous Li distribution inside the Li_1.4_Ni_0.5_Mn_0.5_O_2+_*_x_* lattice during the electrochemically driven intercalation–deintercalation processes. At potentials higher than 4.5 V, the current increase could result from the partial oxidation of the electrolyte upon cycling.

**Figure 5:**
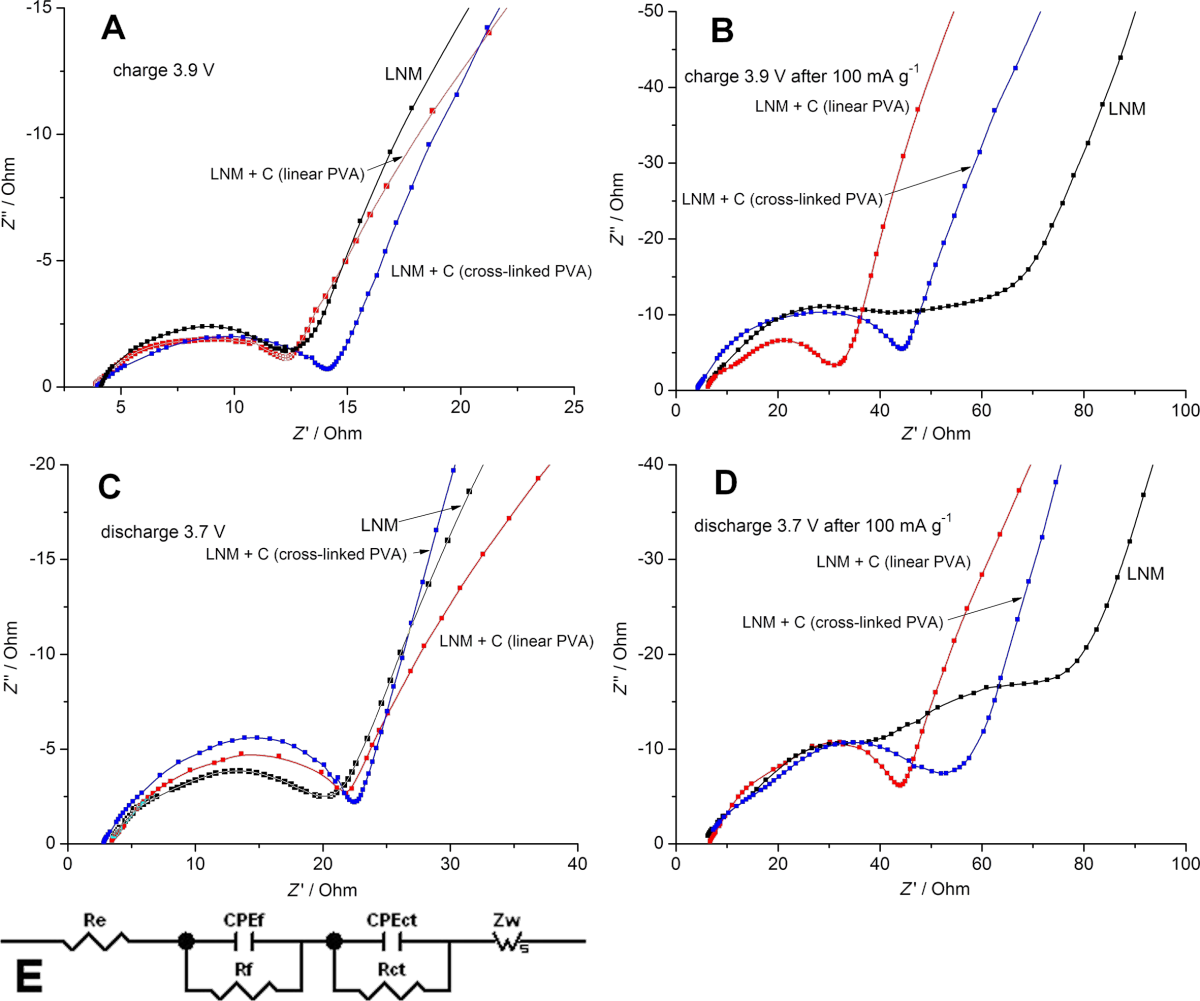
Nyquist plots of pure Li_1.4_Ni_0.5_Mn_0.5_O_2+_*_x_* and LNM/C nanocomposites at the charge potential of 3.9 V: A) 1st cycle, B) after cycling at discharge currents of 20–100 mA·g^−1^ (15th cycle); at the discharge potential of 3.7 V: С) 1st cycle, D) after cycling at discharge currents of 20–100 mA·g^−1^ (15th cycle). E) Equivalent circuit used for fitting the EI spectra.

Electrochemical impedance (EI) measurements were performed to investigate the details of the lithium insertion-extraction processes in LNM/C nanocomposite cathodes (Figure 5A–D). All the plots are mainly composed of a small intercept at high frequencies, a semicircle at high to medium frequencies and a linear part in the low-frequency region. The real and complex EI parts are normalized to the real surface area of each electrode calculated according to the procedure described earlier [31]. The impedance spectra are fitted using the equivalent circuit model (Figure 5E) [34–35]. The small intercept is almost the same for the electrodes and corresponds to the solution resistance of the cell (Re). Rf and CPEf stand for the Li^+^ migration resistance and the capacity of the surface layer, respectively. Rct and *C*PEct stand for the related charge-transfer resistance and the double-layer capacitance, respectively. Zw represents the diffusion-controlled Warburg impedance in the low-frequency region [34–35].

Concerning the 1st cycle, the values of Rct are almost identical for pure Li_1.4_Ni_0.5_Mn_0.5_O_2+_*_x_* and LMN/C nanocomposites (Figure 5А,С). However, after cycling at 20–100 mA·g^−1^, for pure Li_1.4_Ni_0.5_Mn_0.5_O_2+_*_x_* Rct substantially increases in comparison with LNM/C composites (Figure 5B,D). One can suppose that the carbon additives lead to an enhancement of the electronic conductivity of Li_1.4_Ni_0.5_Mn_0.5_O_2+_*_x_* and hinder the interaction of Li_1.4_Ni_0.5_Mn_0.5_O_2+_*_x_* particles with the organic electrolyte.

In order to clarify the influence of carbon nanocoating, the apparent Li^+^ diffusion coefficients (*D*) were estimated both for pure Li_1.4_Ni_0.5_Mn_0.5_O_2+_*_x_* and LNM/C nanocomposites. These diffusion coefficients are calculated from EI data using the following equation [36]:


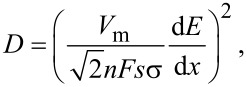


where *n* = 1, σ is the Warburg parameter (Ω·s^−0.5^), *F* is the Faraday constant (96500 C·mol^−1^), *s* is the real surface of the electrode (cm^2^), *V*_m_ is the molar volume of the electrode material (cm^3^·mol^−1^) and d*E*/d*x* is the derivative of the potential versus lithium content.

**Figure 6:**
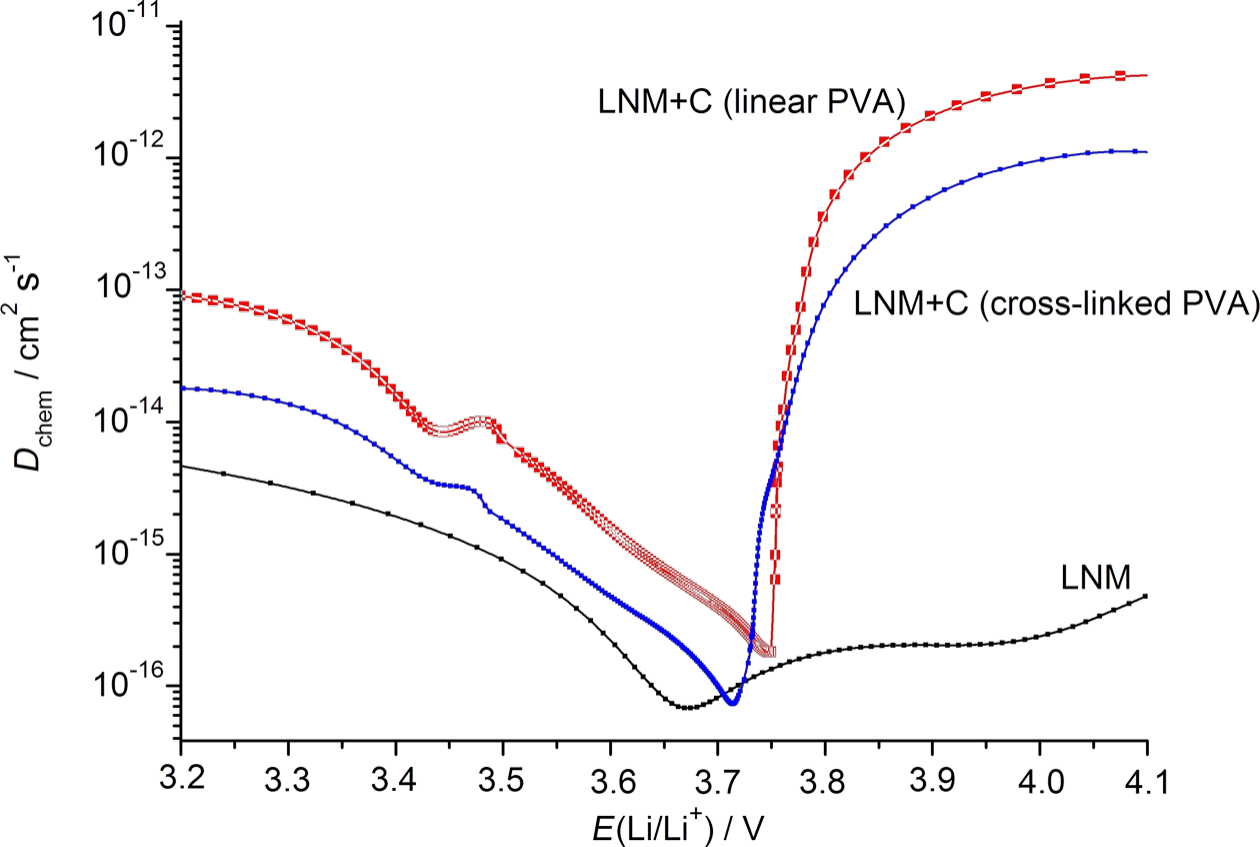
Apparent lithium diffusion coefficients as functions of the discharge potential for pure Li_1.4_Ni_0.5_Mn_0.5_O_2+_*_x_* and LNM/C nanocomposites obtained from linear and cross-linked PVA.

While discharging, the values of the apparent diffusion coefficient of lithium ions for the pure Li_1.4_Ni_0.5_Mn_0.5_O_2+_*_x_* are in the range from 10^−17^ to 10^−15^ cm^2^·s^−1^ with a minimal value of 6.8 × 10^−17^ cm^2^·s^−1^ at 3.67 V (Figure 6). Concerning the LNM/C nanocomposites, the variations of *D* with the applied potential are substantially different. The *D* values are higher practically over the whole potential range, and at potentials higher than 3.75 V they exceed the ones for pure Li_1.4_Ni_0.5_Mn_0.5_O_2+_*_x_* by three orders of magnitude. Most likely, the 3D carbon framework decreases the polarization and the charge-transfer resistance, which promotes the faster Li transport through the electrode–electrolyte interface. The variations of *D* of both LNM/C nanocomposites are rather similar, which points to resemblances in the Li^+^ intercalation processes. The minimal values of *D* are 6.4 × 10^−16^ cm^2^·s^−1^ for the LNM/C composite obtained from linear PVA and 7.3 × 10^−17^ cm^2^·s^−1^ for the one obtained from cross-linked PVA at 3.75 and 3.72 V, respectively. This difference can be explained by the different morphologies and compositions of carbon-based nanocoatings, in particular, by the higher ratio of sp^2^- to sp^3^-hybridized carbon in the LNM/C composite obtained from linear PVA [28]. This results in a higher electronic conductivity and higher *D* values. The different amounts of residual intermediates of pyrolysis also play a role.

**Figure 7:**
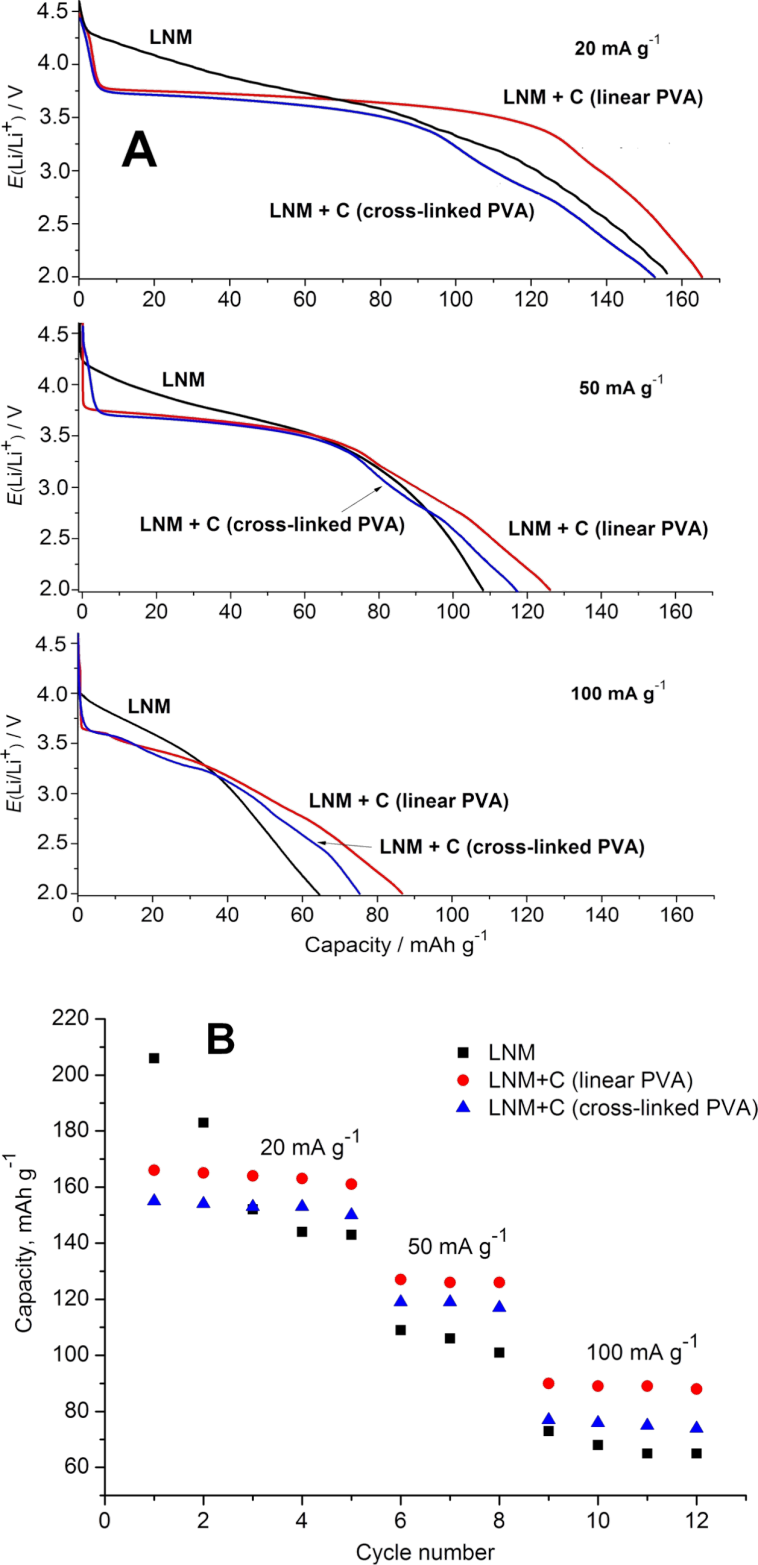
Discharge curves (А) and specific capacities (В) of pure Li_1.4_Ni_0.5_Mn_0.5_O_2+_*_x_* and LNM/C nanocomposites at discharge currents of 20–100 mA·g^−1^.

For all the investigated materials, the charge–discharge curves for the intermediate cycles obtained at different rates are shown in Figure 7A. In contrast to pure Li_1.4_Ni_0.5_Mn_0.5_O_2+_*_x_*, both LNM/C nanocomposites exhibit is a well-pronounced plateau at 3.75 V in the discharge curves, especially at a current density of 20 mA·g^−1^. Apparently, this fact confirms the polarization decrease for the LNM/C composites discussed earlier during the analysis of CVA results.

The discharge capacity variations upon cycling are represented in Figure 7B. In the case of pure Li_1.4_Ni_0.5_Mn_0.5_O_2+_*_x_*, the capacity rapidly drops at the 4th cycle. For the LNM/C nanocomposites, the initial capacity values are lower but the stability upon cycling is significantly better. For the LNM/C nanocomposite obtained from linear PVA, the capacity values are usually higher than for the one obtained from cross-linked PVA. They attain a value of 165 mAh·g^−1^ at a current density of 20 mA·g^−1^. This fact correlates quite well with the higher electronic conductivity of this material and the higher values of the Li^+^ diffusion coefficient.

**Figure 8:**
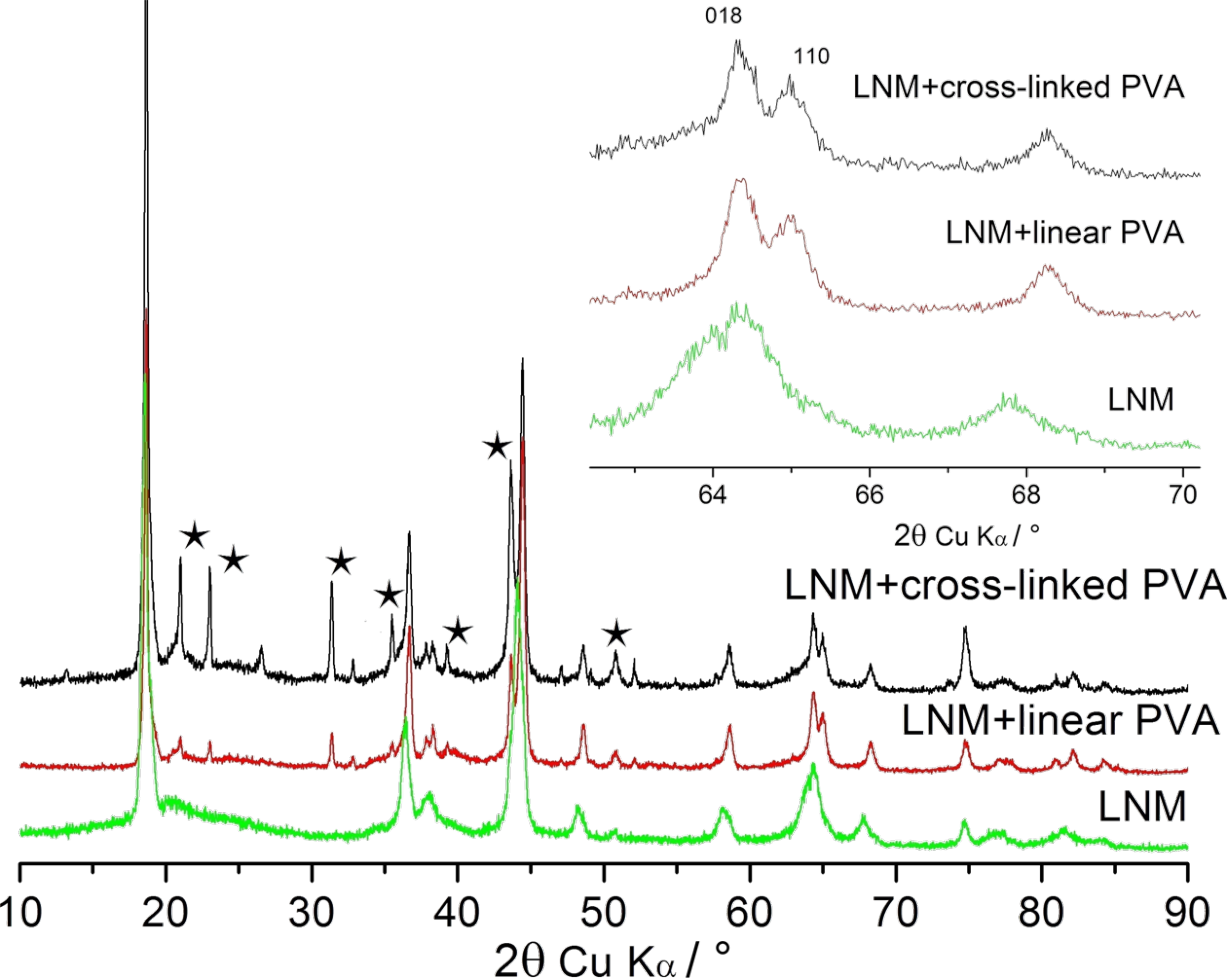
XRD patterns of pure and carbon-coated Li_1.4_Ni_0.5_Mn_0.5_O_2+_*_x_* after electrochemical cycling at 20–100 mAh·g^−1^. The asterisks denote reflections of Li_2_CO_3_ (PC PDF 22-1141).

Figure 8 displays the XRD patterns of electrode materials after electrochemical cycling at high discharge currents. It can be seen that the splitting of (018)/(110) reflections is much higher in carbon-coated materials compared to the pure Li_1.4_Ni_0.5_Mn_0.5_O_2+_*_x_* (Figure 8, inset).

**Figure 9:**
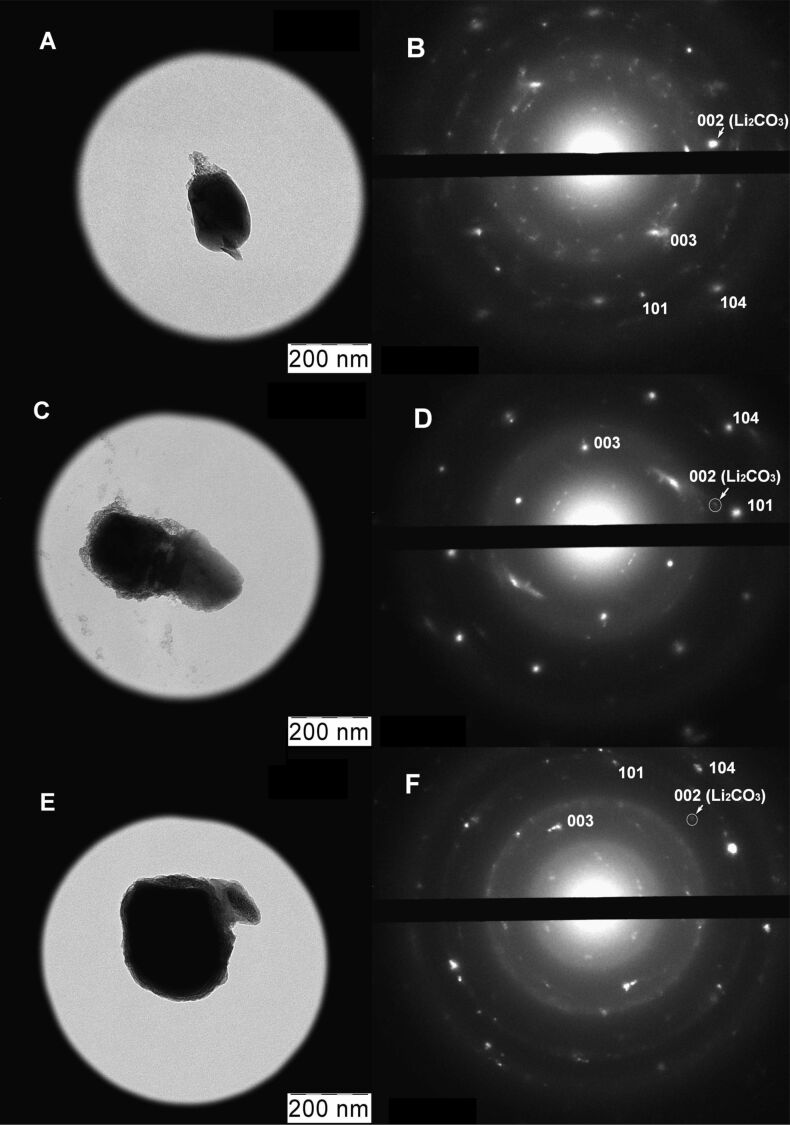
TEM micrographs and SAED patterns of the cycled electrode materials based on Li_1.4_Ni_0.5_Mn_0.5_O_2+_*_x_* (A,B) and LNM/C nanocomposites obtained by the pyrolysis of linear (C,D) and cross-linked (E,F) PVA. The cycling conditions are shown in Figure 7B.

According to the selected area electron diffraction (SAED) patterns, the preservation of the hexagonal structure of Li_1.4_Ni_0.5_Mn_0.5_O_2+_*_x_* is especially visible in the carbon-coated material obtained from linear PVA (Figure 9B).

At high potentials during cycling, the contact of Li_1.4_Ni_0.5_Mn_0.5_O_2+_*_x_* with electrolyte results in an interaction leading to the formation of a Li-deficient spinel Li*_x_*(Ni_0.5_Mn_0.5_)_2_O_4_, and, later, cubic (Ni_0.5_Mn_0.5_)O [37]. Other authors observed a slow transformation of the hexagonal phase into a cubic phase in the course of electrochemical cycling of LiMeO_2_ [38–39]. This process was also accompanied by a systematic decrease in (018)/(110) splitting. The observed processes of the deep crystallochemical degradation of pure Li_1.4_Ni_0.5_Mn_0.5_O_2+_*_x_* (Figure 8) lead to kinetic obstacles to the solid-state diffusion of Li^+^ in Li_1.4_Ni_0.5_Mn_0.5_O_2+_*_x_* crystallites and, hence, to a substantial decrease in its electrochemical capacity values.

The electrode–electrolyte interface in composite electrode materials is reduced by the thin carbon nanocoating that protects a part of Li_1.4_Ni_0.5_Mn_0.5_O_2+_*_x_* surface from electrochemical degradation. SAED patterns show the presence of Li_2_CO_3_ in all cycled samples, although the intensity of the (002) reflections of lithium carbonate is rather weak (Figure 9B,D,F). However, X-ray diffraction patterns demonstrate the presence of a detectable amount of lithium carbonate only in the cycling products of composite electrodes (Figure 8).

Taking into account the lack of other traces of Li_1.4_Ni_0.5_Mn_0.5_O_2+_*_x_* degradation in the C-coated samples, the formation of Li_2_CO_3_ in nanocomposites could be attributed to an interaction of the electrolyte rather with the carbon coating than with the bare Li_1.4_Ni_0.5_Mn_0.5_O_2+_*_x_*. The better electrochemical performance of nanocomposite electrodes during cycling indicates that the formation of Li_2_CO_3_ particles and other related products has a negligible effect on the electrochemical performance of the carbon-coated electrode materials.

Hence, it was demonstrated that even a partial modification of the synthesis technique (additional stage of thermal cross-linking of PVA) leads to LNM/C composites with different properties of the carbon coatings, including a different micro-/nanomorphology and a different ratio of sp^2^- to sp^3^-hybridized carbon. The last feature is also confirmed by the different integral intensities of D- and G-peaks in the Raman spectra of the pyrolysis products of linear and cross-linked PVA [28,40]. The variation of sp^2^- and sp^3^-hybridized carbon content has to do with the features of the composition and pyrolysis of organic polymer precursors. During the pyrolysis of linear PVA at 200–230 °C, its dehydration is accompanied by the formation of C=C chains with conjugated double-bond systems. In the case of cross-linked PVA, the number of free OH-groups is substantially lower due to the formation of chemical bonds between neighboring linear PVA chains. Hence in the course of the dehydration of this polymer, the formation of C=C double bonds is much less probable.

## Conclusion

The various properties of carbonaceous nanocoatings affect both the kinetics of lithium insertion–extraction and the electrochemical properties of LNM/C composites. The presence of carbon results in a decrease of the cell polarization and the charge transfer resistance. This causes an increase of the specific capacity in comparison with pure Li_1.4_Ni_0.5_Mn_0.5_O_2+_*_x_*. Analyzing the CVA, EI and galvanostatic cycling data for LNM/C composites obtained using linear and cross-linked PVA, it can be seen that the presence of a carbon nanocoating and/or a mesoporous carbon framework does not alter the mechanism of lithium insertion–extraction into the Li_1.4_Ni_0.5_Mn_0.5_O_2_ structure but improves its kinetic and electrochemical properties. It is shown that even the nanosized layer of carbon complicates the transformation of hexagonal Li_1.4_Ni_0.5_Mn_0.5_O_2+_*_x_* into the polymorphic cubic structure during cycling through the limitation of electrochemical degradation of Li_1.4_Ni_0.5_Mn_0.5_O_2+_*_x_* at the electrode–electrolyte interface. In the case of linear PVA, LNM/C nanocomposites with a more homogeneous carbon distribution and a higher content of sp^2^-hybridized carbon were obtained. The synergistic effect of these features of ultrathin carbon coatings results in higher values of the lithium diffusion coefficient and a lower charge-transfer resistance of the whole nanocomposite and, hence, to the enhancement of specific capacity of as-obtained cathode material at moderate discharge rates.

## Experimental

The mixed hydroxide (Ni_0.5_Mn_0.5_)(ОН)*_n_*·*x*H_2_O was precipitated from a solution containing Ni and Mn acetates (*С* ≈ 1.5 М) at a given molar ratio by adding 1 М NaOH aqueous solution at room temperature. The precipitates were filtered, repeatedly washed, then placed into a freeze-dryer chamber (Labconco Freezone 7948030 (USA)) and subjected to freeze-drying (*P* = 0.1–0.3 mbar, *T* = −40 °C to +30 °C, 72 h). The obtained mixed hydroxide powders were annealed at 500 °С for 2 h, then mixed with LiOH·H_2_O (≥99%, Fluka) (molar ratio Li/(Ni + Mn) = 1.4) and pressed into pellets. The pellets were subsequently annealed at 500 and 900 °С for 4 h in air at each temperature.

As-prepared Li_1.4_Ni_0.5_Mn_0.5_O_2+_*_x_* powders were mixed with 15 wt % of linear or cross-linked PVA (*M*_r_ = (5–200) × 10^3^, Grade 16/1, Reachim). The cross-linked PVA was obtained by thermal processing of linear PVA according to the procedure described in [26]. The mixtures of Li_1.4_Ni_0.5_Mn_0.5_O_2+_*_x_* and PVA were heated argon up to 350 °С with subsequent dwelling for 15 min, then they were cooled down to room temperature at a rate of 5 °C·min^−1^ in argon [25]. Due to the different character of the pyrolysis processes of linear and cross-linked PVA, the amount of PVA was optimized in order to ensure the comparable carbon content in the final composites (4 and 5.5 wt % for the linear and cross-linked PVA, respectively [28]).

The samples were characterized by XRD using a D/MAX 2500 diffractometer (Rigaku) in the reflection mode with Cu Kα radiation and a curved-graphite [2] monochromator placed in the reflected beam (2θ range 10–90°, step 0.02°, acquisition time 3 s per step).The analysis of diffraction patterns was performed by using WinXPow software and PDF-2 powder diffraction database.

The thermal analysis of the linear and cross-linked PVA was performed in argon by heating to 700 °C at a rate of 10 °C·min^−1^ (STA 209 PC Luxx thermal analyzer (Netzsch)).

The morphology of the composites and their electron diffraction patterns were analyzed by transmission electron microscopy (Libra 200 MC, Carl Zeiss) at an accelerating voltage of 200 kV and a magnification of 30,000–300,000×.

The X-ray photoelectron spectra were acquired with a Kratos Ultra DLD spectrometer using a monochromatic Al Kα X-ray source that possesses an analysis area of 300 μm × 700 μm. The spectra were recorded in a constant analyzer pass energy mode of 5 eV resulting in a resolution better than 0.3 eV.

For the preparation of the electrodes, the cathode paste (active material (85 wt %), acetylene black (10 wt %, Timcal) and polyvinylidene fluoride (5 wt %, solved in *N*-methyl-2-pyrrolidone) was coated on a stainless steel net (0.05 mm thick). After pressing, the electrodes were dried in vacuum (0.5–1.0 mbar) at 120 °C for 8 h. The active electrode, counter electrode (Li) and reference electrode (Li) were placed in a hermetic Teflon cell using a porous polypropylene separator. 1 M LiPF_6_ solution in ethylene carbonate (EC)–dimethyl carbonate (DMC)–diethyl carbonate (DEC) (1:1:1 by volume) was used as an electrolyte. According to Fischer titration data, the water content in the electrolyte did not exceed 25 ppm. In the course of cell assembly, the water content in the glove box did not exceed 0.5 ppm. All the potential values in this manuscript are referred to the Li/Li^+^ electrode.

The galvanostatic curves and cyclic voltammograms (CVA) were registered using 100N Metrohm Autolab equipment. The cells were cycled in a potential range of 2–4.6 V at a current density of 20–100 mA·g^−1^ at room temperature. The potential scan rate was 50 µV·s^−1^.

Electrochemical impedance (EI) measurements were performed on a “Solartron 1255B” using the cells assembled as described above. The amplitude of the AC signal was 5 mV over a frequency range from 1 MHz to 10 mHz. The Corrware 2 and CorrView 2 software (Scribner Associates) was employed for the electrochemical experiments and the obtained hodographs were treated using “ZView-Impedance Software”.
